# Structured headache services as the solution to the ill-health burden of headache. 3. Modelling effectiveness and cost-effectiveness of implementation in Europe: findings and conclusions

**DOI:** 10.1186/s10194-021-01305-8

**Published:** 2021-08-11

**Authors:** Michela Tinelli, Matilde Leonardi, Koen Paemeleire, Alberto Raggi, Dimos Mitsikostas, Elena Ruiz de la Torre, Timothy J. Steiner, on behalf of the European Brain Council Value of Treatment Headache Working Group, the European Headache Federation, the European Migraine and Headache Alliance and Lifting The Burden: the Global Campaign against Headache

**Affiliations:** 1 Care Policy Evaluation Centre, The London School of Economics and Political Science, Houghton Street, London, WC2A 2AE UK; 2 Fondazione IRCCS Istituto Neurologico Carlo Besta, Milan, Italy; 3 Department of Neurology, Ghent University Hospital, Ghent, Belgium; 4 1st Department of Neurology, Aeginition Hospital, Medical School, National and Kapodistrian University of Athens, Athens, Greece; 5 European Migraine and Headache Alliance, Brussels, Belgium; 6 Department of Neuromedicine and Movement Science, Norwegian University of Science and Technology, Trondheim, Norway; 7 Division of Brain Sciences, Imperial College London, London, UK

**Keywords:** Headache, Migraine, Tension-type-headache (TTH), Medication-overuse-headache (MOH), Structured headache services, Health economics, Cost-effectiveness, Quality improvement, Healthy-life-years (HLYs), Global campaign against headache

## Abstract

**Background:**

There have been several calls for estimations of costs and consequences of headache interventions to inform European public-health policies. In a previous paper, in the absence of universally accepted methodology, we developed headache-type-specific analytical models to be applied to implementation of structured headache services in Europe as the health-care solution to headache. Here we apply this methodology and present the findings.

**Methods:**

Data sources were published evidence and expert opinions, including those from an earlier economic evaluation framework using the WHO-CHOICE model. We used three headache-type-specific analytical models, for migraine, tension-type-headache (TTH) and medication-overuse-headache (MOH). We considered three European Region case studies, from Luxembourg, Russia and Spain to include a range of health-care systems, comparing current (suboptimal) care versus target care (structured services implemented, with provider-training and consumer-education). We made annual and 5-year cost estimates from health-care provider and societal perspectives (2020 figures, euros). We expressed effectiveness as healthy life years (HLYs) gained, and cost-effectiveness as incremental cost-effectiveness-ratios (ICERs; cost to be invested/HLY gained). We applied WHO thresholds for cost-effectiveness.

**Results:**

The models demonstrated increased effectiveness, and cost-effectiveness (migraine) or cost saving (TTH, MOH) from the provider perspective over one and 5 years and consistently across the health-care systems and settings. From the societal perspective, we found structured headache services would be economically successful, not only delivering increased effectiveness but also cost saving across headache types and over time. The predicted magnitude of cost saving correlated positively with country wage levels. Lost productivity had a major impact on these estimates, but sensitivity analyses showed the intervention remained cost-effective across all models when we assumed that remedying disability would recover only 20% of lost productivity.

**Conclusions:**

This is the first study to propose a health-care solution for headache, in the form of structured headache services, and evaluate it economically in multiple settings. Despite numerous challenges, we demonstrated that economic evaluation of headache services, in terms of outcomes and costs, is feasible as well as necessary. Furthermore, it is strongly supportive of the proposed intervention, while its framework is general enough to be easily adapted and implemented across Europe.

**Supplementary Information:**

The online version contains supplementary material available at 10.1186/s10194-021-01305-8.

## Introduction

Many studies, in Europe and elsewhere, have shown that headache disorders are under-diagnosed and under-treated (*eg*, [1]). Despite the existence of a range of effective therapies [2], these do not reach large numbers of people who might benefit, or do so inefficiently, delivered by health-care providers without the requisite understanding of these disorders [3]. The solution – structured headache services based in primary care and supported by training and education [3, 4], in a model that is readily adaptable across settings and health-care systems – was described in the first paper in this series [5]. In a later paper, in the absence of universally accepted methodology, we developed headache-type-specific analytical models to be applied to economic evaluation of the model, implemented in three countries in the European Region [6]. Here we apply that methodology, and present the findings.

Indirect costs are a key issue in economic evaluation. Because headache disorders are disabling [7–9], lost productivity is an important consequence, at demonstrably high cost [10–13]. Later papers in this series assess the complex relationship between headache-attributed disability and lost productivity, and consider whether, and to what degree, alleviating the former will lead to recovery of the latter [14, 15]. Our evaluation here allows for the possibility that headache-attributed disability explains only part of lost productivity.

## Methods

The methods are described in detail in the earlier paper [6]. We modelled cost-effectiveness of structured headache services delivering treatments, with efficacies known from randomised controlled trials, for each of migraine, tension-type headache (TTH) and medication-overuse headache (MOH). We did this in the settings of three European Region countries, Russia, Spain and Luxembourg, with differing health-care systems but for which we had population-based data [16–18]. For the two alternatives of current (suboptimal) care and target care (structured services implemented, with provider-training and consumer-education), economic modelling incorporated patient outcomes and cost estimates over two separate timeframes: one and 5 years.

Outcomes were measured in healthy life years (HLYs), and effectiveness as HLYs gained by change from current to target care. We assumed that target care would partially but not entirely close treatment gaps [5]: provider-training within the structured services model would increase coverage and consumer-education would enhance adherence, each, conservatively, by 50% of the gap between current and target care.

Costs included health-care costs (medicines, GP and specialist visits, and examinations) from the provider perspective; additionally, lost productivity (days lost from work) was included in estimates made from the societal perspective. Methodological details are provided elsewhere on the decision-analytical models, on epidemiological data (including disability), estimations of intervention effectiveness, economic outcomes (including use of resources and lost productivity), treatment management plans and selection of interventions for migraine, TTH and MOH within the alternatives under comparison [6].

Economic and effectiveness outcomes were brought together to evaluate cost-effectiveness in terms of costs to be invested per HLY gained (incremental cost-effectiveness ratio [ICER]), with the three health-care systems of Russia, Luxemburg and Spain bringing different systems of health-care service delivery and financing into the model.

Limited evidence supports opportunity-cost–based cost-effectiveness thresholds applicable across diverse countries, including those of interest here. We applied WHO’s thresholds against gross domestic product (GDP) for this purpose: interventions costing < 3*GDP per capita per HLY gained were cost-effective, those costing < 1*GDP per capita per HLY gained were highly cost-effective [19]. Although these lack specificity for any country’s particular contexts, they are the thresholds used by policy makers, who do so in the light of these contexts. Since the overall balance of evidence suggests that WHO’s thresholds may be too high [20], we performed sensitivity analyses to calculate how much we should inflate the costs (or deflate the gains) to meet such thresholds.

The principal analyses were conducted from the health-care provider perspective, with robustness tested in a series of sensitivity analyses inflating health-care costs and deflating HLYs gains while keeping to the same cost-effectiveness thresholds. In a series of secondary analyses, we considered the larger societal perspective. For the baseline societal analysis, we assumed all lost productivity was explained by disease-attributed disability, whereas, in a conservative alternative, we assumed that this disability accounted for only 20% of lost productivity (so that only this proportion might be recovered).

## Results

**Fig. 1:**
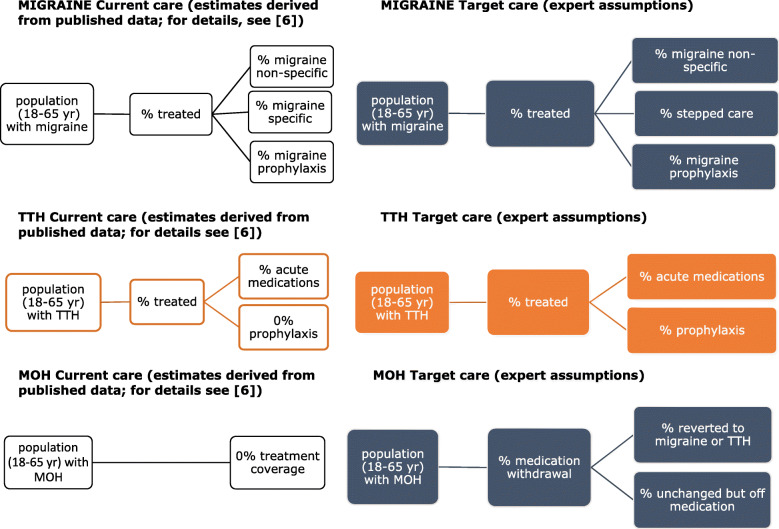
Analytical models according to headache type

Summaries of the economic and effectiveness outcomes for the treatments of each headache type are presented in Tables 1 (Luxembourg), 2 (Russia) and 3 (Spain). Analytical models according to headache type are reported in Fig. 1.

**Table 1:** Luxembourg: economic consequences of changing from current to target care (population estimates)

	Numbers of patients	124,713	127,501	14,378
**1-YEAR TIME FRAME**		**MIGRAINE**	**TTH**	**MOH**
Health-care provider perspective	Additional costs (euros)	2,468,610	(−58,977,322) (cost saved)	(− 304,638) (cost saved)
	HLYs gained	1126	51	776
	ICER (euros spent for each HLY gained)	2192	n/a	n/a
5-YEAR TIME FRAME		**MIGRAINE**	**TTH**	**MOH**
Health-care provider perspective	Additional costs (euros)	8,148,427	(−59,712,128) (cost saved)	(−1,423,598) (cost saved)
	HLYs gained	5265	239	3625
	ICER (euros spent for each HLY gained)	1548	n/a	n/a

**Table 2:** Russia: economic consequences of changing from current to target care (population estimates)

	Numbers of patients	18,122,512	26,679,239	7,193,081
**1-YEAR TIME FRAME**		**MIGRAINE**	**TTH**	**MOH**
Health-care provider perspective	Additional costs (euros)	215,273,678	(−80,743,387) (cost saved)	(−81,939,062) (cost saved)
	HLYs gained	163,709	10,695	388,112
	ICER (euros spent for each HLY gained)	1315	n/a	n/a
**5-YEAR TIME FRAME**		**MIGRAINE**	**TTH**	**MOH**
Health-care provider perspective	Additional costs (euros)	1,066,657,492	(− 153,433,010) (cost saved)	(− 382,907,727) (cost saved)
	HLYs gained	765,026	49,964	1,813,677
	ICER (euros spent for each HLY gained)	1394	n/a	n/a

**Table 3:** Spain: economic consequences of changing from current to target care (population estimates)

	Numbers of patients	10,772,263	7,850,265	2,128,185
**1-YEAR TIME FRAME**		**MIGRAINE**	**TTH**	**MOH**
Health-care provider perspective	Additional costs (euros)	216,491,177	(−63,402,506) (cost saved)	(− 49,026,722) (cost saved)
	HLYs gained	97,311	3146	114,829
	ICER (euros spent for each HLY gained)	2225	n/a	n/a
**5-YEAR TIME FRAME**		**MIGRAINE**	**TTH**	**MOH**
Health-care provider perspective	Additional costs (euros)	688,382,902	(−122,434,131) (cost saved)	(− 229,105,755) (cost saved)
	HLYs gained	454,741	14,702	536,604
	ICER (euros spent for each HLY gained)	1514	n/a	n/a

**Fig. 2:**
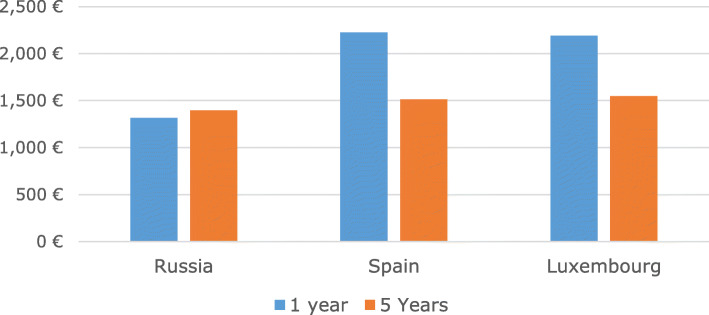
Economic analysis: Incremental cost-effectiveness ratio (euros spent for each HLY gained) at one year and 5 years, from health-provider perspective (migraine). Note: For tension-type headache and medication-overuse headache, the intervention is not only more cost-effective than current care but also cost saving over 1 and 5 years (see Tables 1, 2 and 3)

In short-term modelling (1-year time frame) from the health-care provider perspective, the intervention was found to be cost-effective for migraine (Fig. 2) – well below WHO thresholds [19] – and cost saving for TTH and MOH (see Tables 1, 2 and 3). Over 5 years the intervention appeared even more cost-effective for migraine (Fig. 2) and cost-saving for TTH and MOH (the amounts of costs saved are reported in Tables 1, 2 and 3). Sensitivity analyses showed the robustness of these findings (Additional file 1: Appendices 1–3). For example, for Russia and migraine, the intervention was still cost-effective after inflating health-care costs – or deflating effectiveness gains – by factors of 100 (Additional file 1: Appendix 2).

From the health-care provider perspective, the hypothetical shift to target care would bring gains in HLYs (the longer the time frame the greater the gain). For migraine, resources must be invested to secure these benefits (the longer the time frame, the lower, relatively, the investment). For TTH and MOH, the benefits would be accompanied by cost savings (the longer the time frame the greater the economic gain). Findings were consistent across the health-care systems of the three countries.

From the societal perspective (Additional file 1: Appendices 4–9), the intervention was not only more effective than current care, but also cost saving – for all headache types, across health-care systems and in both 1-year and 5-year time frames. In the conservative scenario, where remedying disability would recover only 20% of lost productivity, the intervention remained cost-effective across all models.

Finally, we considered cost and effectiveness outcomes for 1000 patients with migraine, 1000 with TTH and 1000 with MOH patients in each country setting, allowing comparisons of the impact of introducing target care across country-specific populations (Additional file 1: Appendices 7–9). The greater the country’s wage levels, the greater were the economic savings for society (*ie*, Luxembourg > Spain > Russia).

## Discussion

For the first time, effectiveness and cost-effectiveness of introducing structured headache services have been evaluated. Our results show, across three diverse health-care systems in European Region, that structured headache services based in primary care and supported by consumer-education and provider-training [5] are an effective and economically viable solution to headache disorders and the disability they cause. From the health-care provider perspective, TTH services are not only cost-effective, but also cost-saving (ICERs negative). Although this disorder is associated with much lower estimates of health loss [7–9] than migraine or MOH, structured headache services will not discriminate: they are not selective, and must manage all headache types. In practice, people with TTH are least likely to require these services, while the consumer-education component of structured services would be expected to reduce doctor visits for TTH and save health-care resources.

Lost productivity weighs heavily in economic estimates. The savings in work productivity modelled in our study were greater than the investments in health care estimated to meet these savings (a finding predicted long ago by WHO [3]). For TTH, the saving was more evident in Luxembourg, because of its higher wage-levels [10].

Of course, these findings assumed that lost productivity reported as a consequence of headache would, therefore, be recovered commensurately as headache was alleviated. There was reason to doubt this assumption, since many extraneous factors influence the relationship between headache-attributed disability and lost productivity [14, 15]. It is arguable that these factors are much more constant at individual level [15], so that the assumption might hold, but this is untestable. Instead, we relied on sensitivity analyses, in which the intervention remained cost-effective across all models even with the alternative conservative assumption that alleviating headache would recover only 20% of the lost productivity attributed to it.

There have been repeated calls for better modelling of costs and outcomes of headache interventions to inform public-health policies, given the very high prevalence of headache disorders [7, 21–23]. There is no widely accepted framework to help European (or other) countries undertake economic evaluation of headache interventions in order to establish which alternative(s) provides the best value for money. Indeed, this topic seems perversely under-researched given the much-increased awareness of the global burden of headache [2, 3, 7–9]. To the best of our knowledge, ours is the first study to provide such a framework, and, crucially, it has not confined itself to specific individual treatments but evaluated a health-care delivery package offering a range of treatments. Our work is still incomplete: much remains to be done, particularly in pilot implementations of structured headache services to gather empirical evidence to support our currently hypothetical findings. In the meantime, we have demonstrated that systematic evaluation of headache-type-specific outputs and costs of headache services is feasible (as well as necessary), while work progresses on service quality evaluation [24–28], also of high importance if services are to be implemented.

The principal limitations of this study were those inherent in economic modelling. We were dependent on the type and quality of the data sourced in order to calculate the economic outcomes, the latter being imperfect in Eurolight [29]. Similar limitations applied to the effectiveness outcomes. We made many assumptions in the costing model [6], and could anticipate that our findings would be sensitive to variations in these, but countered this by conducting sensitivity analyses. Although cost-effectiveness thresholds used routinely by WHO (and applied in our analysis) have been criticized for being too high [20], our results appear robust and generally undercut these thresholds.

## Conclusions

Even with very conservative assumptions, highly inflating costings (or deflating expected gains), we could conclude that structured headache services would be cost-effective according to WHO thresholds [19] – and this held true for all headache types and across all settings.

The framework of the proposed intervention is general enough to be easily adapted and implemented [5]. Thus, structured headache services offer an efficient, equitable, effective and cost-effective solution to headache, a cause of much population ill health [12, 13, 16] and heavy economic burden [23].

Structured headache services – offering care efficiently and equitably to the widest number of people [5] and, according to our findings here, an economically viable solution to headache as a cause of public ill health – are in accord with WHO’s vision of universal health coverage (UHC) [30]. The concept of UHC is that all people should have access to the health services they need, when and where they need them, without financial hardship. UHC is based on strong, people-centred primary health care, while good health systems are rooted in the communities they serve. Care models like structured headache services that define a clear primary-care role [5] and allow economic evaluation promote the goal of UHC worldwide.

## Supplementary Information

**Additional file 1: Appendix 1.** Baseline vs sensitivity analyses: Luxembourg. **Appendix 2.** Baseline vs sensitivity analyses: Russia. **Appendix 3.** Baseline vs sensitivity analyses: Spain. **Appendix 4.** Luxembourg: economic results of changing from current to target care (population estimates). **Appendix 5.** Russia: economic results of changing from current to target care (population estimates). **Appendix 6.** Spain: economic results of changing from current to target care (population estimates). **Appendix 7.** Differences in outcomes when changing from current to target care (cohorts of 1000 patients per type of headache in Luxembourg). **Appendix 8.** Differences in outcomes when changing from current to target care (cohorts of 1000 patients per type of headache in Russia). **Appendix 9.** Differences in outcomes when changing from current to target care (cohorts of 1000 patients per type of headache in Spain).

## Data Availability

All data generated or analysed during this study are included in this published article.

## Abbreviations

GDP: Gross domestic product
HLY: Healthy Life Year
ICERs: Incremental cost-effectiveness ratios
MOH: Medication-overuse headache
TTH: Tension-type headache
UHC: Universal health coverage
WHO: World Health Organization

## References

[1] Katsarava Z, Mania M, Lampl C, Herberhold J, Steiner TJ (2018). Poor medical care for people with migraine in Europe - evidence from the Eurolight study. J Headache Pain.

[2] Steiner TJ, Jensen R, Katsarava Z, Linde M, EA MG, Osipova V, Paemeleire K, Olesen J, Peters M, Martelletti P, on behalf of the European Headache Federation and Lifting The Burden: the Global Campaign against Headache (2019). Aids to management of headache disorders in primary care (2nd edition). J Headache Pain.

[3] World Health Organization, Lifting The Burden (2011). Atlas of headache disorders and resources in the world 2011.

[4] Steiner TJ, Antonaci F, Jensen R, Lainez JMA, Lantéri-Minet M, Valade D, on behalf of the European Headache Federation and Lifting The Burden: the Global Campaign against Headache (2011). Recommendations for headache service organisation and delivery in Europe. J Headache Pain.

[5] Steiner TJ, Jensen R, Katsarava Z, Stovner LJ, Uluduz D, Adarmouch L, Al Jumah M, Al Khathaami AM, Ashina M, Braschinsky M, Broner S, Eliasson JH, Gil-Gouveia R, Gómez-Galván JB, Guðmundsson LS, Kawatu N, Kissani N, Kulkarni GB, Lebedeva ER, Leonardi M, Linde M, Luvsannorov O, Maiga Y, Milanov I, Mitsikostas DD, Musayev T, Olesen J, Osipova V, Paemeleire K, Peres MFP, Quispe G, Rao GN, Risal A, Ruiz de la Torre E, Saylor D, Togha M, Yu SY, Zebenigus M, Zenebe Zewde Y, Zidverc-Trajković J, Tinelli M, on behalf of Lifting The Burden: the Global Campaign against Headache (2021) Structured headache services as the solution to the ill-health burden of headache. 1: rationale and description. J Headache Pain 22 (to be added in proof) 10.1186/s10194-021-01265-z PMC8293530 34289806

[6] Tinelli M, Leonardi M, Paemeleire K, Mitsikostas D, de la Torre ER, Steiner TJ, on behalf of the European Brain Council Value of Treatment Headache Working Group, the European Headache Federation, the European Federation of Neurological Associations, and Lifting The Burden: the Global Campaign against Headache (2021) Structured headache services as the solution to the ill-health burden of headache. 2: modelling effectiveness and cost-effectiveness of implementation in Europe. Methodol J Headache Pain 22 (to be added in proof)

[7] GBD 2016 Neurology collaborators (2019). Global, regional, and national burden of neurological disorders, 1990-2016: a systematic analysis for the global burden of disease study 2016. Lancet Neurol.

[8] GBD 2017 Disease and injury incidence and prevalence collaborators (2018). Global, regional, and national incidence, prevalence, and years lived with disability for 354 diseases and injuries for 195 countries and territories, 1990-2017: a systematic analysis for the global burden of disease study 2017. Lancet.

[9] GBD 2019 Viewpoint collaborators (2020). Five insights from the global burden of disease study 2019. Lancet.

[10] Linde M, Gustavsson A, Stovner LJ, Steiner TJ, Barré J, Katsarava Z, Lainez JM, Lampl C, Lantéri-Minet M, Rastenyte D, Ruiz de la Torre E, Tassorelli C, Andrée C (2012). The cost of headache disorders in Europe: the Eurolight project. Eur J Neurol.

[11] Ayzenberg I, Katsarava Z, Sborowski A, Obermann M, Chernysh M, Osipova V, Tabeeva G, Steiner TJ (2015). Headache yesterday in Russia: its prevalence and impact, and their application in estimating the national burden attributable to headache disorders. J Headache Pain.

[12] Stewart WF, Ricci JA, Chee E, Morganstein D, Lipton R (2003). Lost productive time and cost due to common pain conditions in the US workforce. JAMA.

[13] Simić S, Rabi-Žikić T, Villar JR, Calvo-Rolle JL, Simić D, Simić SD (2020). Impact of individual headache types on the work and work efficiency of headache sufferers. Int J Environ Res Public Health.

[14] Kothari SF, Jensen RH, Steiner TJ (2021) The relationship between headache-attributed disability and lost productivity. 1. A review of the literature. J Headache Pain 22 (in press) 10.1186/s10194-021-01264-0 PMC8285879 34273952

[15] Thomas H, Kothari SF, Husøy A, Jensen RH, Katsarava Z, Tinelli M, Steiner TJ (2021) The relationship between headache-attributed disability and lost productivity. 2. Empirical evidence from population-based studies in 11 countries. J Headache Pain 22 (in press) 10.1186/s10194-021-01362-z PMC8903529 34922442

[16] Ayzenberg I, Katsarava Z, Sborowski A, Chernysh M, Osipova V, Tabeeva G, Yakhno N, Steiner TJ (2012). The prevalence of primary headache disorders in Russia: a countrywide survey. Cephalalgia.

[17] Ayzenberg I, Katsarava Z, Sborowski A, Chernysh M, Osipova V, Tabeeva G, Steiner TJ (2014). Headache-attributed burden and its impact on productivity and quality of life in Russia: structured healthcare for headache is urgently needed. Eur J Neurol.

[18] Steiner TJ, Stovner LJ, Katsarava Z, Lainez JM, Lampl C, Lantéri-Minet M, Rastenyte D, Ruiz de la Torre E, Tassorelli C, Barré J, Andrée C (2014). The impact of headache in Europe: principal results of the Eurolight project. J Headache Pain.

[19] World Health Organization (2014) Choosing Interventions that are Cost–Effective (WHO-CHOICE). Project WHO | WHO-CHOICE. WHO Available at https://www.who.int/news-room/feature-stories/detail/new-cost-effectiveness-updates-from-who-choice (Accessed 27 July 2021)

[20] Woods B, Revill P, Sculpher M, Claxton K (2016). Country-level cost-effectiveness thresholds: initial estimates and the need for further research. Value Health.

[21] Stovner LJ, Hagen K, Jensen R, Katsarava Z, Lipton RB, Scher AI, Steiner TJ, Zwart J-A (2007). The global burden of headache: a documentation of headache prevalence and disability worldwide. Cephalalgia.

[22] Viana M, Khaliq F, Zecca C, Figuerola MDL, Sances G, Di Piero V, Petolicchio B, Alessiani M, Geppetti P, Lupi C, Benemei S, Iannacchero R, Maggioni F, Jurno ME, Odobescu S, Chiriac E, Marfil A, Brighina F, Barrientos Uribe N, Pérez Lago C, Bordini C, Lucchese F, Maffey V, Nappi G, Sandrini G, Tassorelli C (2020). Poor patient awareness and frequent misdiagnosis of migraine: findings from a large transcontinental cohort. Eur J Neurol.

[23] Steiner TJ, Stovner LJ, Vos T, Jensen R, Katsarava Z (2018). Migraine is first cause of disability in under 50s: will health politicians now take notice?. J Headache Pain.

[24] Peters M, Jenkinson C, Perera S, Loder E, Jensen R, Katsarava Z, Gil Gouveia R, Broner S, Steiner T (2012). Quality in the provision of headache care. 2: defining quality and its indicators. J Headache Pain.

[25] Katsarava Z, Gil Gouveia R, Jensen R, Gaul C, Schramm S, Schoppe A, Steiner TJ (2015). Evaluation of headache service quality indicators: pilot implementation in two specialist-care centres. J Headache Pain.

[26] Schramm S, Uluduz D, Gil Gouveia R, Jensen R, Siva A, Uygunoglu U, Gvantsa G, Mania M, Braschinsky M, Filatova E, Latysheva N, Osipova V, Skorobogatykh K, Azimova J, Straube A, Emre Eren O, Martelletti P, De Angelis V, Negro A, Linde M, Hagen K, Radojicic A, Zidverc-Trajkovic J, Podgorac A, Paemeleire K, De Pue A, Lampl C, Steiner TJ, Katsarava Z (2016). Headache service quality: evaluation of quality indicators in 14 specialist-care centres. J Headache Pain.

[27] Pellesi L, Benemei S, Favoni V, Lupi C, Mampreso E, Negro A, Paolucci M, Steiner TJ, Ulivi M, Cevoli S, Guerzoni S (2017). Quality indicators in headache care: an implementation study in six Italian specialist-care centres. J Headache Pain.

[28] Steiner TJ, Göbel H, Jensen R, Lampl C, Paemeleire K, Linde M, Braschinsky M, Mitsikostas D, Gil-Gouveia R, Katsarava Z, on behalf of the European Headache Federation and Lifting The Burden: the Global Campaign against Headache (2019). Headache service quality: the role of specialized headache centres within structured headache services, and suggested standards and criteria as centres of excellence. J Headache Pain.

[29] Andrée C, Stovner LJ, Steiner TJ, Barre J, Katsarava Z, Lainez JM, Lair M-L, Lanteri-Minet M, Mick G, Rastenyte D, Ruiz de la Torre E, Tassorelli C, Vriezen P, Lampl C (2011). The Eurolight project: the impact of primary headache disorders in Europe. Description of methods J Headache Pain.

[30] World Health Organization (2020). Universal health coverage.

