# Supplementary material for: Structured headache services as the solution to the ill-health burden of headache. 3. Modelling effectiveness and cost-effectiveness of implementation in Europe: findings and conclusions
Source: J Headache Pain. 2021 Aug 11;22(1):90. doi: 10.1186/s10194-021-01305-8 (PMC8359596; doi:10.1186/s10194-021-01305-8)
Supplement: Supplementary file 1 — Additional file 1: Appendix 1. Baseline vs sensitivity analyses: Luxembourg. Appendix 2. Baseline vs sensitivity analyses: Russia. Appendix 3. Baseline vs sensitivity analyses: Spain. Appendix 4. Luxembourg: economic results of changing from current to target care (population estimates). Appendix 5. Russia: economic results of changing from current to target care (population estimates). Appendix 6. Spain: economic results of changing from current to target care (population estimates). Appendix 7. Differences in outcomes when changing from current to target care (cohorts of 1000 patients per type of headache in Luxembourg). Appendix 8. Differences in outcomes when changing from current to target care (cohorts of 1000 patients per type of headache in Russia). Appendix 9. Differences in outcomes when changing from current to target care (cohorts of 1000 patients per type of headache in Spain). [file 10194_2021_1305_MOESM1_ESM.docx]

**Appendix 1: Baseline *vs* sensitivity analyses: Luxembourg**

|  |  |
| --- | --- |
|  |  |

Please note that for tension-type headache the intervention is not only more effective than current care but also cost saving – see negative ICERs. It remains cost saving even when inflating the costs 100 times. GDP = 88,165uros (World Bank data); interventions costing <88,165 euros per capita per HLY gained (blue line) are highly cost-effective; interventions costing <3x88,165 euros per capita per HLY gained (orange line) are cost-effective.

**Appendix 2: Baseline *vs* sensitivity analyses: Russia**

|  |  |
| --- | --- |

Please note that for tension-type headache the intervention is not only more effective than current care but also cost saving – see negative ICERs. It remains cost saving even when inflating the costs 100 times. GDP = 7,666 euros (World Bank data); interventions costing <7,666 euros per capita per HLY gained (blue line) are highly cost-effective; interventions costing <3x7,666 euros per capita per HLY gained (orange line) are cost-effective.

**Appendix 3: Baseline *vs* sensitivity analyses: Spain**

|  |  |
| --- | --- |

Please note that for tension-type headache the intervention is not only more effective than current care but also cost saving – see negative ICERs. It remains cost saving even when inflating the costs 100 times. GDP = 23,292 euros (World Bank data); interventions costing <23,292 euros per capita per HLY gained (blue line) are highly cost-effective; interventions costing <3x23,292 euros per capita per HLY gained (orange line) are cost-effective.

**Appendix 4: Luxembourg: economic results of changing from current to target care (population estimates)**

| 1-YEAR TIME FRAME |  | MIGRAINE | TTH | MOH |
| --- | --- | --- | --- | --- |
|  | **Numbers of patients** | **124,713** | **127,501** | **14,378** |
| Societal perspective | Cost saved (euros) | (-41,812,553) | (-72,328,350) | (-61,785,377) |
|  | HLYs gained | 1,126 | 51 | 776 |
| Societal perspective (disability accounts for 20% of lost productivity) | Cost saved (euros) | (-6,387,623) | (-61,647,528) | (-12,357,075) |
|  | HLYs gained | 1,126 | 51 | 776 |
| 5-YEAR TIME FRAME |  | **MIGRAINE** | **TTH** | **MOH** |
| Societal perspective | Cost saved (euros) | (-198,780,953) | (-122,102,542) | (-288,727,962) |
|  | HLYs gained | 5,265 | 239 | 3,625 |
| Societal perspective (disability accounts for 20% of lost productivity) | Cost saved (euros) | (-33,237,449) | (-72,190,217) | (-57,745,592) |
|  | HLYs gained | 5,265 | 239 | 3,625 |

**Appendix 5: Russia: economic results of changing from current to target care (population estimates)**

| 1-YEAR TIME FRAME |  | MIGRAINE | TTH | MOH |
| --- | --- | --- | --- | --- |
|  | **Numbers of patients** | **18,122,512** | **26,679,239** | **7,193,081** |
| Societal perspective | Cost saved (euros) | (-476,646,793) | (-392,518,661) | (-3,562,419,117) |
|  | HLYs gained | 163,709 | 10,692 | 388,112 |
| Societal perspective (disability accounts for 20% of loss productivity) | Cost saved (euros) | 122,783,947  (cost incurred) | (-132,268,993) | (-712,483,823) |
|  | HLYs gained | 163,709 | 10,692 | 388,112 |
| 5-YEAR TIME FRAME |  | **MIGRAINE** | **TTH** | **MOH** |
| Societal perspective | Cost saved (euros) | (-2,434,826,670) | (-1,673,642,150) | (-16,647,466,708) |
|  | HLYs gained | 765,026 | 49,964 | 1,813,677 |
| Societal perspective (disability accounts for 20% of loss productivity) | Cost saved (euros) | 366,360  (cost incurred) | (-457,474,838) | (-3,329,493,341) |
|  | HLYs gained | 765,026 | 49,964 | 1,813,677 |

**Appendix 6: Spain: economic results of changing from current to target care (population estimates)**

| 1-YEAR TIME FRAME |  | MIGRAINE | TTH | MOH |
| --- | --- | --- | --- | --- |
|  | **Numbers of patients** | **10,772,263** | **7,850,265** | **2,128,185** |
| Societal perspective | Cost saved (euros) | (-1,298,706,730) | (-389,046,356) | (-3,619,390,406) |
|  | HLYs gained | 97,311 | 3,146 | 114,829 |
| Societal perspective (disability accounts for 20% of loss productivity) | Cost saved (euros) | (-86,548,404) | (-128,531,276) | (-723,878,081) |
|  | HLYs gained | 97,311 | 3,146 | 114,829 |
| 5-YEAR TIME FRAME |  | **MIGRAINE** | **TTH** | **MOH** |
| Societal perspective | Cost saved (euros) | (-6,392,256,936) | (-1,644,193,634) | (-16,913,698,056) |
|  | HLYs gained | 454,742 | 14,702 | 536,604 |
| Societal perspective (disability accounts for 20% of loss productivity) | Cost saved (euros) | (-727,745,066) | (-426,786,031) | (-3,382,739,611) |
|  | HLYs gained | 454,742 | 14,702 | 536,604 |

**Appendix 7: Differences in outcomes when changing from current to target care (cohorts of 1,000 patients per type of headache in Luxembourg)**

|  |  |
| --- | --- |
|  |  |
|  |  |

**Health-care provider perspective - cost (euros); Societal perspective - cost (euros);
 Societal perspective (disability accounts for 20% of lost productivity) - cost (euros); HLYs gained**

**Appendix 8: Differences in outcomes when changing from current to target care (cohorts of 1,000 patients per type of headache in Russia)**

|  |  |
| --- | --- |
|  |  |
|  |  |

**Health-care provider perspective - cost (euros); Societal perspective - cost (euros);
 Societal perspective (disability accounts for 20% of lost productivity) - cost (euros); HLYs gained**

**Appendix 9: Differences in outcomes when changing from current to target care (cohorts of 1,000 patients per type of headache in Spain)**

|  |  |
| --- | --- |
|  |  |

**Health-care provider perspective - costs (euros); Societal perspective - cost (euros);
 Societal perspective (disability accounts for 20% of lost productivity) - cost (euros); HLYs gained**
